# Epigenetics and Asthma: A Systematic Review

**DOI:** 10.7759/cureus.71052

**Published:** 2024-10-08

**Authors:** Mohammed Dauwood Aslam, Ahmed Ageed

**Affiliations:** 1 General Practice, Health Education West Yorkshire, Huddersfield, GBR; 2 Medicine and Surgery, University Hospitals of Leicester NHS Trust, Leicester, GBR

**Keywords:** allergen, antibodies, asthma, dna methylation, epigenetics, histone deacetylation, immunity, inflammatory response, t-lymphocytes

## Abstract

Asthma is a chronic respiratory condition characterized by the narrowing of the airways, causing difficulty in breathing. Its heritability has long been an area of research, and the study of genetics alone has not been sufficient in the explanation of both its heritability and susceptibility. The study of epigenetics, which is defined as "the study of heritable changes in gene expression that do not involve changes to the underlying DNA sequence," can be used to explain the heritability and susceptibility of asthma. These epigenetic alterations to our DNA are influenced by environmental factors which are already known as risk factors for asthma; these include factors such as diet, smoking, and air pollution. The epigenetic mechanisms work by altering gene transcription and therefore determine whether specific genes are expressed, such mechanisms include DNA methylation and histone acetylation.

## Introduction and background

Asthma is a chronic inflammatory disease of the lungs that causes bronchial spasms and tightness of the airways upon exposure to allergens, leading to breathlessness and wheezing. The severity and regularity of asthma differ depending on the individual, ranging from hourly to daily [1]. In the UK alone, asthma is a condition that 5.4 million people are getting treatment for, 20% of whom are children [2]. Asthma is the cause of death in around 1000 people per annum, 900 of those deaths are associated with avoidable factors [2]. Therefore, the importance of understanding how this disease is inherited is crucial for the medical community so that this knowledge can be used to diagnose, treat, and prevent the condition.

There is an implication for a significant genetic contribution to the development of asthma. This is seen in the studies of asthma inheritance in twins. Studies have revealed that there is an increased concordance rate for monozygotic twins (0.74) than dizygotic twins (0.35), therefore suggesting that there is a strong genetic component to asthma inheritance [3]. This inheritance, however, is a subject that has not been clarified satisfactorily through the studies of Mendelian inheritance alone as there are also environmental factors that contribute to its development. The interaction between genes and the environment is linked by the study of epigenetics which looks at how both environmental and genetic components alter gene expression. Epigenetics is a term defined as "the study of heritable changes in gene expression that do not involve changes to the underlying DNA sequence." These changes in gene expression are controlled by a group of chemical compounds known as the ‘epigenome,' which controls which genes in the genome are expressed [4]. Epigenomic compounds work by attaching to DNA and altering its function. When this is done, the DNA is said to be ‘marked.' There are many different epigenetic modifications including DNA/RNA protein interactions, however, this report will focus on the two main types of epigenetic markers, the first being DNA methylation (DNAM) and the second being histone acetylation.

Epigenetic alterations occur naturally through normal cellular development and differentiation; however, this process is also influenced by responses to environmental factors. Living in a developed country has been shown as a strong risk factor for asthma [5]. This increased risk may be associated with increased exposure to outdoor allergens such as microbial and viral pathogens, airborne particulates, ozone, diesel exhaust particles (DEP), pollens, outdoor molds, environmental tobacco smoke (ETS), cold air, and humidity [6]. The way the epigenome responds to these factors can be damaging to the individual's health which is why they are termed as ‘risk factors'. This paper shall discuss how these risk factors affect the epigenome and contribute to the inheritance of asthma.

## Review

Method

**Figure 1:**
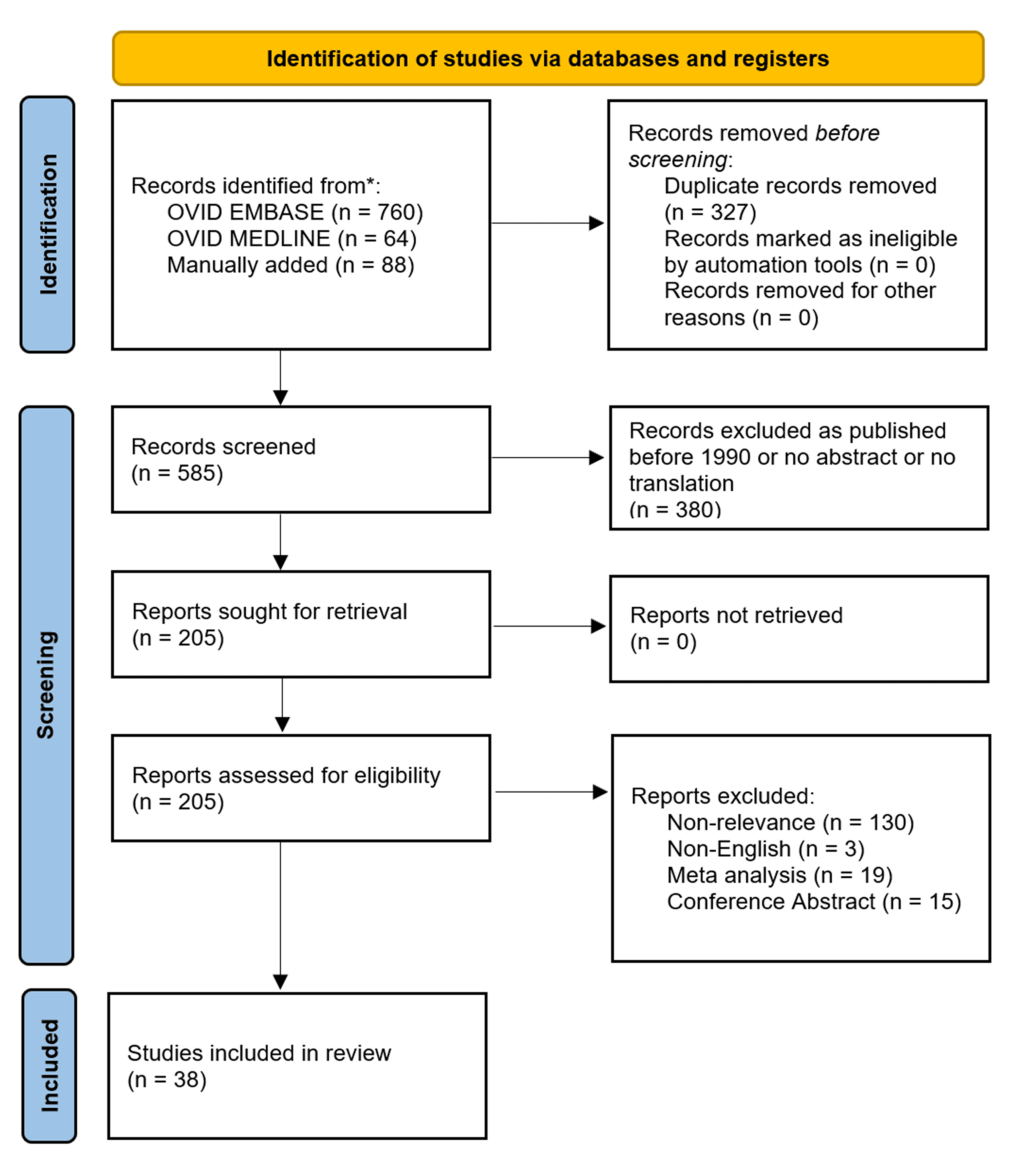
PRISMA Flow Chart Diagram PRISMA: Preferred Reporting Items for Systematic Reviews and Meta-Analyses

This study adhered to the guidelines set forth by the Preferred Reporting Items for Systematic Reviews and Meta-Analyses (PRISMA) Figure 1 [7]. Several electronic databases were utilized for our search, including Ovid Embase and Ovid Medline. Searches were limited to documents published in English and were further categorized by geographical focus to more economically developed countries. Our search terms were "Asthma", "Epigenetics", and "Immunity", along with keywords like hypersensitivity and allergy. We employed a combination of these search terms and keywords using "AND" and "OR" as Boolean operators. Additionally, we examined the reference lists of all relevant articles to identify further studies. The references were exported to EndNote for duplicate removal, resulting in 585 references to be screened. The studies were then evaluated for eligibility, with the criteria specifying that only articles in English and published after 1990 were included. Additionally, the articles had to be original studies, excluding meta-analyses and conference abstracts. After exclusion, the total number of studies that were selected was 38.

DNA methylation 

DNA methylation is an epigenetic mechanism that affects gene expression by inhibiting transcription, thus resulting in gene silencing. The way DNA methylation occurs is by adding a methyl (CH3) group onto the 5th carbon atom of the cytosine ring, forming 5-methylcytosine (5-mC), which is found in approximately 1.5% of genomic DNA [8]. 5-mC appears predominantly at CpG sites; this is a location where cytosine and guanine bases are seen sequentially on a DNA sequence in the 5' to 3' direction, however, the observation of 5-mC is not limited to this location only. 5-mC is also seen in non-CpG positions in embryonic stem cells [9]. The majority of CpG sites are methylated, though there are some unmethylated CpG sites. DNA methylation prevents gene expression from occurring as the methyl groups block enzymes that are responsible for transcription from attaching to the DNA. The unmethylated CpG sites are clustered into CpG islands (sites of CpG clusters), this is very important as this allows gene expression to occur, however when these CpG islands become methylated, then the expression of the gene is switched off [10].

The attachment of methyl groups is regulated and undertaken by a group of enzymes known as DNA methyltransferases (DNMTs). When DNA replication occurs, the enzyme DNA helicase will separate the double helix into two separate strands, each carrying one methylated cytosine causing the daughter DNA duplex to be hemi-methylated, leaving only one strand methylated correctly. There are three major classes of DNMTs: DNMT1, DNMT3a & DNMT3b. The role of DNMT1 is to maintain already established patterns of DNA methylation; it does this by methylation of the hemimethylated DNA and is therefore informally termed the maintenance DNMT. DNMT3a and 3b, however, are known as de novo DNMTs, as they establish new patterns of DNA methylation [9].

DNMT 1 is termed the maintenance DNMT as it continues the trend of methylation from the parent DNA to the newly formed strands of DNA. When DNA replication occurs, the parent DNA is methylated however the newly formed strands are not. DNMT 1 works by binding onto hemimethylated DNA at CpG sites and adds a methyl group to the cytosine of the newly synthesized strand, therefore maintaining the previously established CpG methylation pattern.

DNMT3a and DNMT3b, however, work differently as they bind to both hemimethylated and non-methylated DNA, this form of DNA methylation is termed de novo methylation [9]. This occurs after the embryo has been implanted and is crucial for primary development, a lack of these DNMTs can lead to crucial consequences such as immunodeficiency-centromeric instability-facial anomalies syndrome (ICF), a rare autosomal recessive condition seen in patients with a mutation in DNMT3b [11].

When looking into asthma specifically, a study was conducted that revealed an upregulation of the expression of suppressors of cytokine signalling3 (Socs3) in a mouse model of asthma. Ultimately, DNMTs are the controllers of the epigenome as it is through their regulation of DNA transcription that determines whether genes are or are not expressed. The study found that there were low levels of DNMT1 in the mouse model which may induce the overexpression of Socs3 and inhibit a family of compounds responsible for repressing the expression of genes known as the HDAC family. Given these findings, a study of DNMT1 and its effects on asthma epigenetics could lead to the making of new drugs against asthma [12].

Environmental factors such as allergens, air pollution, and tobacco smoke significantly influence DNA methylation in asthma patients. These factors can alter the epigenetic landscape, particularly by disrupting DNA methylation patterns in genes related to inflammation and immune response. For example, exposure to air pollutants like particulate matter (PM2.5) has been shown to increase oxidative stress, leading to changes in the DNA methylation of airway cells, which can exacerbate asthma symptoms by influencing gene expression related to inflammation and airway remodeling [13]. Similarly, allergens and smoking may also lead to hypomethylation of certain immune-related genes, further contributing to asthma development and severity [14].

Histone remodeling

The other epigenetic mechanism responsible for increasing gene transcription is histone acetylation. This works by altering the capability of transcriptional regulatory enzymes and transcription factors to coordinate with DNA and with each other to produce the right transcriptional response [15]. The role of histone proteins is to package DNA so that it manages to fit within the parameters of the nucleus into structural units called nucleosomes. For gene expression to occur, however, these tightly packed meters of DNA need to unwind for them to be accessible for binding by activating factors, which occurs through chromatin remodeling; this works by rearranging the physical state of chromatin from a condensed state to one that is accessible for transcription [10].

The enzymes responsible for histone acetylation are called histone acetyltransferases (HATs). These enzymes are crucial in the controlling of histone acetylation for the core histone proteins H3 and H4. When in the ‘closed’ state, the DNA is tightly wrapped around these histones, thus preventing the enzyme RNA polymerase II from binding to the DNA, thus activating messenger RNA (mRNA) for transcription. The acetylation of these histones forms chemical tags that recruit other transcriptional complexes and chromatin remodeling compounds which allow for the loosening of the nucleosome thus allowing RNA polymerase to bind to the naked DNA and activate mRNA to allow gene transcription to take place [16]. The genes that are not transcribed as often have repressive marks, these genes are packed more tightly meaning that their ability to undergo transcription is less as there is less access to the DNA for transcription to take place [15].

The mechanism of removing acetyl groups from histones is called histone deacetylation. This process is carried out by an enzyme called histone deacetylase (HDAC), which comes in 18 different forms in humans and is also known as the ‘epigenetic eraser" [17]. These remove the acetyl groups from histones causing repackaging of chromatin, therefore, reducing transcription levels and therefore gene expression levels. Histone acetylation, therefore, is dependent on the balance between HDAC activity which inhibits acetylation, resulting in gene silencing, and HAT activity which promotes acetylation and gene expression. An imbalance of these enzymes has been associated with cancer progression as well as tumorigenesis [10].

There is an alteration in the levels of histone deacetylases in asthma patients compared to normal patients. In the bronchial biopsies in asthma patients, the levels of HAT in the airways is increased, therefore leading to raised inflammatory gene expression [18]. There is also a similar change noticed in the alveolar macrophages of asthmatic patients with a reduction in HDAC1 expression, which would also lead to raised inflammatory gene expression due to decreased transcriptional inhibition [19]. It is worthy to note that the activity of HAT and HDAC in the peripheral lymphocytes and monocytes were normal, suggesting that the changes that occur in asthma patients are only acting locally on the airways. As well as this, it was noted in bronchial biopsies that patients who were smokers and also asthmatic had a more significant reduction of HDAC activity [18], which is why smoking is known as a risk factor of the disease, the effects of which shall be discussed in more detail later in this report.

Epigenetic regulation of the immune response

Asthma, amongst all other allergies, is a type 1 IgE-mediated hypersensitivity reaction [20]. Hypersensitivity reactions are brought about by specific antigens called allergens, this induces responses such as the maturation of B cells and the production of antibodies and memory B cells [21]. Type 1 hypersensitivity is unique in that it produces IgE antibodies as opposed to IgM and IgG as seen in the normal humoral immune response. The receptors that IgE binds to are called Fc receptors which are located on granulocytes. Upon binding to these receptors, the granulocytes become sensitized which causes cross-linking of the IgE on the surface of the cells [22]. This cross-linking initiates a positive feedback loop through the release of inflammatory mediators, including bronchoconstrictor agents, into the surrounding tissues, which exacerbates the allergic response and contributes to asthma symptoms [22].

The development of asthma is dependent on the differentiation of naïve CD4+ T lymphocytes into the TH2 lineage [23]. These TH2 cells cause the B cells to respond to the antigens thus instigating antibody production, which further perpetuates the immune response in a positive feedback loop. This differentiation pathway is partly dependent upon epigenetic factors. The promoter regions of the CpG sites contained within naïve CD4+ cells are methylated both in the IL-4 and Interferon (IFN)- γ genes, which are important cytokines in the lineage of T cells [24]. When CD4+ T lymphocytes are activated by allergens, the levels of demethylation in the IL-4 gene increase, and there is also a correlation between the amount of loss of methylation and IL-4 expression [24].

For inflammation to be induced, many signaling pathways must be activated, particularly the nuclear factor kappa B (NF-𝜿B) pathway which is active in all the variant forms of asthma [25,26]. Alterations to the epigenetic profile occur upon NF-𝜿B binding onto inflammatory gene promoters and the introduction of inflammatory mediators [25,26]. These epigenetic altercations are mainly seen in the differences in histone acetylation. These changes play a significant role in the induction of many pro-inflammatory genes in the smooth muscle and epithelial cells of the lungs [27]. The release of TNF-α stimulated eotaxin (a form of chemokine) is linked with the binding of NF-𝜿B as well as histone H4 acetylation in airway smooth muscle. This mechanism contributes to a positive feedback loop, where increased histone acetylation is associated with heightened inflammation [27]. 

However, there is also a negative feedback loop that helps regulate this process. IFN- γ can inhibit the TNF-α initiated expression of NF-𝜿B sensitive genes as well as eotaxin [28]. The IFN- γ reduced the amount of HAT activity and increased the HDAC activity. The inhibitory effect of IFN-γ is prevented through the HDAC inhibitor TSA. Therefore, there is no inhibition of gene expression induced by TNF-α. This means that it is the activity of HDAC alongside expression in airway smooth muscle cells that mediates the inhibition of chemokine and cytokine expression induced by TNF-α [29]. The inhibitory role of IFN-γ demonstrates a negative feedback loop that limits excessive inflammation, helping to maintain some level of control over the immune response.

Ultimately, asthma development involves both positive feedback loops, which drive inflammation and exacerbate symptoms, and negative feedback loops, which act to regulate and limit the inflammatory process. This balance between amplification and inhibition is key to understanding the complex immune responses in asthma.

Epigenetics and the inheritance of asthma 

Epigenetics is partly responsible for the heritability of asthma. Epigenetic changes that occur in parental DNA can be passed down to their offspring through a process called ‘imprinting' [15]. The expression of most genes is an expression of the inheritance of chromosomes from both the mother and father. However, this is not the case with imprinted genes. Imprinted genes work in a ‘parent-of-origin-dependent manner,' meaning that the inheritance of gene expression is not dependent upon the DNA sequence but rather is dependent on whether the gene was inherited from the mother or father [29]. It has been shown in studies that it may be the case that asthma development is determined more by maternal inheritance than paternal inheritance [30]. This statement has been backed by statistics such as the risk of a child under the age of 5 developing asthma is three times greater when associated with maternal asthma as opposed to paternal asthma [31].

Some mechanisms have been put forward to establish the mechanism for asthma inheritance. For example, maternal inheritance is seen in the human leukocyte antigen gene ‘HLA-G' which is involved in the modulation of the immune response [32]. There are links associating HLA-G with asthma; this is seen when analyzing the expression of the gene in children with bronchial hyperresponsiveness. It was discovered that children whose mothers also had bronchial hyperresponsiveness had overexpression of the HLA-G allele compared to those whose mothers were not affected by the condition [32]. While the mechanism of the maternal inheritance in asthma has not yet been officially recognized, there are suggestions that it is due to imprinting that these differences in gene expression from each parent are seen.

Effects of environmental factors on the epigenetic profile 

While monozygotic twins have the same genome, their epigenomes can be significantly different depending on the environmental conditions they are exposed [33]. This is due to the influence that environmental stressors have on the epigenetic profile by altering the transcriptional program of cells [34].

Asthma levels have increased on a global scale in developing countries that are making a transitional change to more Western styles of living. This has been particularly evident in the increase in the incidence of asthma in India where some areas of the country have shown an increase of 50% in the prevalence of asthma whereas other regions which have maintained their traditional cuisine have been less affected [35]. Diet significantly impacts the epigenetic profile such as keeping healthy DNA methylation patterns. There are specific nutrients that aid DNA methylation such as folate and choline which is a source of methyl groups. Studies were conducted on mice that have shown a link between altered levels of folic acid and modified methylation patterns & expression of genes related to allergy [36]. Hollingsworth et al. conducted an experiment that showed that increasing the methyl content in the diet of female mice resulted in an increased level of allergy-induced airway diseases in their children [36]. Certain genes are key in the regulation of the adaptive immune response. When a change in diet altered the methylation of these genes, the expression of these genes was also altered. This resulted in an increased risk of developing allergic airway disease, which includes asthma [36].

The evidence is clear that in-utero exposure to environmental tobacco smoke (ETS) results in an increased risk of poor respiratory function and diseases such as asthma [37]. The correlation between maternal smoking and asthma is thought to be associated with alterations in DNA methylation patterns. However, a causal link has not yet been established [38]. It is proposed that the oxidative stress of DNA methylation is changed by smoking. This alteration affects the binding of DNMTs by stopping them from binding to and causing the methylation of DNA thus leading to hypomethylation [38].

Gene expression is dependent on the balance between the activity of HAT and HDAC which have opposing effects, HAT promoting gene expression and HDAC promoting gene silencing [39]. An epigenetic effect of tobacco smoke is that distorts the homeostasis between these two enzymes present in the airway immune system cells. This was demonstrated in a study that was recently conducted which looked at the comparison between bronchoalveolar lavage alveolar macrophages and biopsy specimens between two different subjects of the same age; the first being healthy non-smokers and the second being healthy tobacco smokers [39]. It was observed that those who smoked tobacco had a reduced level of HDAC activity while their expression of inflammatory mediators (e.g., IL-8 GM-CSF) was increased [6]. Due to the macrophages playing a critical role in regulating allergen-induced airway inflammation, if their epigenetic profile is disturbed by tobacco, then this is likely to be a contributing factor to the development of asthma [39].

Air pollutants are very minute and can, therefore, be deeply inhaled, having significant damaging effects on the airways. Similarly, to smoking, air pollution also has effects on epigenetics by causing hypomethylation due to oxidative stress. Benzene is an air pollutant that has been shown to exhibit an eight-fold increase in the risk of asthma development in children and is also associated with changes in the epigenetic profile by altering DNA methylation patterns [40,41].

Certain air pollutants can reduce the concentration of allergen required to cause a decrease in Forced Expiratory Volume in One Second (FEV_1_) by a specific amount [42]. For example, one study reported that one hour of exposure to ozone (0.12 ppm) caused a reduction in the concentration of grass allergen required to reduce FEV_1_ by twenty percent [42]. This shows that there is clear evidence showing a link between certain air pollutants and the exacerbation of asthma in subjects who already had the disease. The link between air pollution and the actual development of asthma however has varied findings within the literature, with some studies reporting no significant association [43] whereas a recent multi-cohort study has shown that exposure during the first three years of life was associated with increased asthma incidence by early (<5 years) and middle (<12 years) childhood [44].

Limitations 

The searches of this literature review were conducted on two databases, Ovid Medline and Ovid Embase, there was therefore potential for further literature to be found on other databases. There was also limited availability of gold-standard randomized controlled trials relating to epigenetics and asthma specifically. 

## Conclusions

There are various epigenetic mechanisms which have different effects on gene expression. These mechanisms have aided our understanding of the characteristics and inheritance of different diseases, and how they may be brought about through alterations to the epigenetic profile. While there is still not substantial proof that these alterations are the causal link of diseases such as asthma, the epigenetic changes in people who diagnosed with it is clear to see. Further research must be done to clarify whether epigenetics does have a causal link to disease or not. This knowledge can then be used to create new drugs that target these epigenetic abnormalities and convert our understanding of epigenetics into practical solutions for curing asthma.
